# Supplementary material for: Self-propelling and rolling of a sessile-motile aggregate of the bacterium Caulobacter crescentus
Source: Commun Biol. 2020 Oct 16;3:587. doi: 10.1038/s42003-020-01300-w (PMC7568532; doi:10.1038/s42003-020-01300-w)
Supplement: Supplementary file 1 — Supplementary Information [file 42003_2020_1300_MOESM1_ESM.pdf]

Supplementary Information:
Self-propelling and rolling of a sessile-motile aggregate of the bacterium *Caulobacter crescentus*

Yu Zeng[1] and Bin Liu[1]

[1] *Department of Physics, University of California, Merced, Merced 95343*

Supplementary Method 1. Hydrodynamic simulation of rosette kinematics

To investigate the key effects of the flagellar alignment and the nearby solid surface on rosette movements, we considered a simplified hydrodynamic model of a rosette propelled by a single flagellum. The schematic of this model is shown in Supplementary Fig. **3**. Here, we regarded the rosette as a sphere and the flagellum as a helical filament. The alignment of the flagellum relative to the rosette center is characterized by an alignment angle θ_{cf} (Supplementary Fig. **3**). Two extreme cases with $\theta_{cf} = 0$ and $\theta_{cf} = \pi/2$ correspond to the radial and orthoradial flagellar alignments, respectively. For simplicity, we ignored any hydrodynamic interactions between the rosette aggregate and its flagellum, each of which can thus be studied analytically by considering its resistance matrix [1]. In such a resistive-force type treatment, the hydrodynamic force ($\mathbf{F}_c$) and torque ($\mathbf{L}_c$) on the rosette (or the sphere) can be formulated to be proportional to the rosette's linear ($\mathbf{u}$) and angular ($\boldsymbol{\omega}$) velocities as

$$\begin{pmatrix} \mathbf{F}_c \\ \mathbf{L}_c \end{pmatrix} = \mathcal{M}_c \cdot \begin{pmatrix} \mathbf{u} \\ \boldsymbol{\omega} \end{pmatrix}, \tag{S1}$$

where $\mathcal{M}_c$ is a 6×6 resistance matrix determined only by geometries. A similar formulation for the flagellum (or the helix) becomes

$$\begin{pmatrix} \mathbf{F}_f \\ \mathbf{L}_f \end{pmatrix} = \mathcal{M}_f \cdot \begin{pmatrix} \mathbf{u}_f \\ \boldsymbol{\omega}_f \end{pmatrix}, \tag{S2}$$

where $\mathbf{F}_f$, $\mathbf{L}_f$, $\mathbf{u}_f$, $\boldsymbol{\omega}_f$, and $\mathcal{M}_f$ are the corresponding force, torque, linear velocity, angular velocity, and resistance matrix for the flagellum, respectively.

To solve the above equation set for rosette movement, it is convenient to define a body-fixed reference frame (x', y', z') with the helical axis of the flagellum lying in the x' direction (Supplementary Fig. **3**). Given a flagellar motor rotating at a constant speed ω_0, the movements of the rosette and its flagellum satisfies

$$\mathbf{u}_f = \mathbf{u} + \boldsymbol{\omega} \times \overrightarrow{O_c O_f}, \tag{S3}$$

$$\boldsymbol{\omega}_f = \boldsymbol{\omega} + \omega_0 \hat{x}', \tag{S4}$$

where $\overrightarrow{O_c O_f}$ denotes a vector connecting the center of the rosette to the center of the flagellum (Supplementary Fig. **3**). In addition, the rosette-flagellum system must obey the force-balance and moment-balance conditions, i.e.,

$$\mathbf{F}_c + \mathbf{F}_f = \mathbf{0}, \tag{S5}$$

$$\mathbf{L}_c + \mathbf{L}_f + \overrightarrow{O_c O_f} \times \mathbf{F}_f = \mathbf{0}. \tag{S6}$$

We computed the resistance matrix for the rosette ($\mathcal{M}_c$) by BIM, subjected to its distance to the wall d that lies in the x-y plane of a lab frame of reference. The resistance matrix for the flagellum, however, depends not only on the distance d but also on its orientation. This makes the model almost impractical due to infinite possibilities of the flagellum orientation in the lab frame of references. Here, we make use of the fact that the flagellum is typically far from the solid wall, and consider its resistance matrix only in the free-space case. In this way, we computed all elements of this matrix in the body-fixed frame of reference $\mathcal{M}_{f,0}$ [2] and applied it for any orientation through a rotation operation,

$$\mathcal{M}_f = \begin{pmatrix} \mathcal{R} & 0 \\ 0 & \mathcal{R} \end{pmatrix} \cdot \mathcal{M}_{f,0} \cdot \begin{pmatrix} \mathcal{R} & 0 \\ 0 & \mathcal{R} \end{pmatrix}, \tag{S7}$$

Here, $\mathcal{R}$ is a 3D-space rotation matrix that can be obtained by

$$\left(\mathbf{e}_x, \mathbf{e}_y, \mathbf{e}_z\right) = \mathcal{R} \cdot \left(\mathbf{e}_{x'}, \mathbf{e}_{y'}, \mathbf{e}_{z'}\right), \tag{S8}$$

where $\left(\mathbf{e}_x, \mathbf{e}_y, \mathbf{e}_z\right)$ and $\left(\mathbf{e}_{x'}, \mathbf{e}_{y'}, \mathbf{e}_{z'}\right)$ are the coordinate basis for the lab and body-fixed reference frames, respectively.

Given the resistance matrices ($\mathcal{M}_c$ and $\mathcal{M}_f$) and motor speed (ω_0), the movement of the rosette ($\mathbf{u}$, $\boldsymbol{\omega}$) is readily solved by the above equation set. Simulation results for a typical rosette size ($R = 3$ μm) and two extreme cases of flagellar alignment (radial and orthoradial) are presented in Supplementary Movie 5 and Supplementary Movie 6, respectively. Despite the distinct flagellar alignments, both cases converge to a periodic circulation in the same direction (here, CCW from above the solid surface for the CW motor). We can thus measure such circulation direction from our experiment and use it as an indicator for the running state of the flagellar motor (CW or CCW). It should be noted that the periodicity of these circular movements can be potentially violated by the relatively more irregular shape of an actual rosette. In addition, the rotational speed of the rosette does not appear to decrease significantly with the presence of the nearby wall (for a fixed motor speed), which validates the uses of the free-space rosette model for scaling analyses.

Supplementary Method 2. Rotation reconstruction

The 3D rotation of a *Caulobacter crescentus* rosette is reconstructed from the movement of its cross section, captured by a single top-view camera. A z-stack of rosette images (Supplementary Fig. **4**a) was used to reconstruct the rosette radius R (with its distribution shown in Supplementary Fig. **1**). To quantify the rotation of an imaged rosette, we first obtain its projected 2D velocity field by performing particle image velocimetry (PIV) analysis on adjacent frames (Supplementary Fig. **4**b) [3]. We then fit this quasi-2D information by a rigid-body rotation to achieve all x–y–z components of the angular velocity. The fitting algorithm is elaborated in the following.

An array of PIV data points, e.g., (x_i ,y_i ; u_i, v_i) with $i = 1, 2, \cdots N$, subjected to a rigid-body rotation at an angular velocity $\boldsymbol{\omega} = (\omega_x, \omega_y, \omega_z)$, should satisfy

$$\begin{aligned} u_i &\approx u(x_i, y_i) = \omega_y \delta z - \omega_z (y_i - y_0), \\ v_i &\approx v(x_i, y_i) = \omega_z (x_i - x_0) - \omega_x \delta z, \end{aligned} \tag{S9}$$

where (x_0, y_0) is the geometric center, and δz is the axial offset of the imaged object (with lateral position (x_i, y_i)) from the rotation center. The best-fit rotation thus corresponds to the minimization of a loss function

$$S(\boldsymbol{\omega}) = \sum_{i=1}^{N} (u_i - u(x_i, y_i))^2 + \sum_{i=1}^{N} (v_i - v(x_i, y_i))^2 \tag{S10}$$

The angular velocity ω_0 that minimizes this loss function can thus computed by a vanishing gradient:

$$\nabla_{\boldsymbol{\omega}} S(\boldsymbol{\omega})|_{\boldsymbol{\omega}=\boldsymbol{\omega}_0} = \mathbf{0}, \tag{S11}$$

or

$$\omega_{0,z}x_0 + \omega_{0,x}\delta z - \bar{x}\omega_{0,z} = -\bar{v}, \tag{S12}$$

$$\omega_{0,z}y_0 + \omega_{0,y}\delta z - \bar{y}\omega_{0,z} = \bar{u}, \tag{S13}$$

$$\begin{aligned} &\bar{v}x_0 - \bar{u}y_0 - \omega_{0,x}\delta z(\bar{x} - x_0) - \omega_{0,y}\delta z(\bar{y} - y_0) + \\ &[\overline{x^2} + \overline{y^2} + x_0^2 + y_0^2 - 2(x_0\bar{x} + y_0\bar{y})]\omega_{0,z} = \overline{vx} - \overline{uy}, \end{aligned} \tag{S14}$$

where symbol $(\bar{\cdot})$ is the ensemble average, e.g., $\bar{u} = \frac{1}{N}\sum_{i=1}^{N} u_i$, and $\overline{ux} = \frac{1}{N}\sum_{i=1}^{N} u_i x_i$. All three components of $\boldsymbol{\omega}_0$ can be solved by an array of linear equations

$$\begin{pmatrix} \delta z & 0 & x_0 - \bar{x} \\ 0 & \delta z & y_0 - \bar{y} \\ 0 & 0 & \overline{\Delta x^2 + \Delta y^2} \end{pmatrix} \cdot \begin{pmatrix} \omega_{0,x} \\ \omega_{0,y} \\ \omega_{0,z} \end{pmatrix} = \begin{pmatrix} -\bar{v} \\ \bar{u} \\ \overline{vx} - \overline{uy} - \bar{v}\bar{x} + \bar{u}\bar{y} \end{pmatrix}. \tag{S15}$$

However, it should be noted that only the z-component of the angular velocity $\omega_{0,z}$ can be obtained explicitly without any prerequisite knowledge of the geometric center (x_0, y_0) and the axial offset δz of the imaged particles.

To obtain the x–y components of ω_0, we utilize the finite focal depth of the imaging system and consider the variant axial positions δz of the PIV sampling sites. Let $u' = u + \omega_{0,z}(y - y_0)$ and $v' = v - \omega_{0,z}(x - x_0)$, we have

$$\delta z = u'/\omega_y = -v'/\omega_x, \tag{S16}$$

or

$$u'\omega_x + v'\omega_y = 0. \tag{S17}$$

A loss function for $\omega_{0,x-y}$ can thus be formulated as

$$S_{x-y}(\Theta) = \sum_{i=1}^{N}(u_i'\cos\Theta + v_i'\sin\Theta)^2, \tag{S18}$$

where Θ is the angle of $\omega_{0,x-y}$ from the x-axis, i.e., $\tan(\Theta) = \omega_{0,y}/\omega_{0,x}$. Minimizing the loss function gives

$$\left.\frac{\mathrm{d}S_{x-y}(\Theta)}{\mathrm{d}\Theta}\right|_{\Theta=\Theta_0} = \sum_{i=1}^{N} 2\left(u_i'\cos\Theta_0 + v_i'\sin\Theta_0\right)\left(-u_i'\sin\Theta_0 + v_i'\cos\Theta_0\right) = 0. \tag{S19}$$

The angle Θ_0 for the best fitting $\boldsymbol{\omega}_0$ can be obtained by finding the roots of the above equation. Multiple solutions are resolved by identifying the Θ_0 that leads to the global minimum of the loss function $S_{x-y}(\Theta)$. In this way, the orientation of $\boldsymbol{\omega}_0$ in the x–y plane, Θ_0, is well determined by the PIV data. However, the magnitude of its in-plane component $\omega_{0,x-y} = \sqrt{\omega_{0,x}^2 + \omega_{0,y}^2}$ is yet to be determined, which requires further axial information of the velocity field.

We consider the finite z variation of the PIV images to acquire the magnitude of the angular velocity. According to Eq. (S16), the axial offset of the particle imaged at (x_i, y_i) from the rotation center of the rosette is given by

$$\delta z_i = \frac{1}{\omega_{0,x-y}}\left(u_i'\sin\Theta_0 - v_i'\cos\Theta_0\right). \tag{S20}$$

If the fluctuation of δz_i can be measured independently, we can then estimate $\omega_{0,x-y}$ by computing the standard deviation of both sides:

$$\omega_{0,x-y} = \frac{1}{\mathrm{SD}_z}\left[\frac{1}{N}\sum_{i=1}^{N}\left(u_i' \sin\Theta_0 - v_i' \cos\Theta_0 - \overline{u'}\sin\Theta_0 + \overline{v'}\cos\Theta_0\right)^2\right]^{1/2}, \quad \text{(S21)}$$

where SD_z denotes the standard deviation of δz_i in the rosette image.

An example of the reconstructed rotation is shown in Supplementary Fig. **4**c and d. For demonstration purposes, we set the δz variation as $\mathrm{SD}_z = 0.5$ μm (which are further characterized as discussed in the following section). The corresponding δz_i (obtained from Eq. (S20)) is also shown in Supplementary Fig. **4**d.

Supplementary Method 3. Axial (z) variation characterization in a rosette image

In our experiment, the fluctuation of δz_i is due to the finite depth of the imaged zone of the microscope (at 100×). Here, we assume the probability of a particle (located at the height z_i) to be captured by a PIV analysis is proportional to the contrast level of its image. Figure **5** shows the contrast level of a single bacterium in phase-contrast as a function of its height, with the contrast level C defined as $C = \frac{|I_{\text{bacterium}} - I_{\text{surround}}|}{I_{\text{background}}}$, where $I_{\text{bacterium}}$, I_{surround}, and $I_{\text{background}}$ are the average light intensities within the contour of a bacterium, its surrounding neighbor, and the entire image, respectively. We let the probability distribution function of visualizing an object located at z, $P(z)$, be proportional to its contrast level, i.e., $P(z) = C(z)/\int_{-d/2}^{d/2} C(z)\mathrm{d}z$, where d is the sample depth. For a spherical object with radius R, the sample depth $d = 2\sqrt{R^2 - \rho^2}$ with ρ the radius in polar coordinates. The standard deviation SD_z in a rosette image can thus be estimated by

$$\mathrm{SD}_z(R) \approx \left[2\int_0^R \left(\int_{-\sqrt{R^2-\rho^2}}^{\sqrt{R^2-\rho^2}} C(z)z^2\mathrm{d}z \Big/ \int_{-\sqrt{R^2-\rho^2}}^{\sqrt{R^2-\rho^2}} C(z)\mathrm{d}z\right)\rho\mathrm{d}\rho/R^2\right]^{1/2}. \quad \text{(S22)}$$

As shown in Supplementary Fig. **5**a, $C(z)$ can be well defined by a superposition of three gaussian distributions, i.e., $C(z) \propto e^{-\left(\frac{z}{d_1}\right)^2} + a\left(e^{-\left(\frac{z-b}{d_2}\right)^2} + e^{-\left(\frac{z+b}{d_2}\right)^2}\right)$, where $a = 0.68 \pm 0.10$, $b = 5.0 \pm 1.0$ μm, $d_1 = 1.2 \pm 0.5$ μm, and $d_2 = 3.7 \pm 1.1$ μm. The value SD_z can thus be computed as a function of the rosette radius R (Eq. (S22)). As shown in Supplementary Fig. **5**b, SD_z increases monotonically with R, which is consistent with the direct measurement of the fluctuation of the light intensity within a rosette image (inset of Supplementary Fig. **5**b). Given SD_z, the in-plane angular velocity $\omega_{0,x-y}$ for each individual rosette is reconstructed accordingly (Eq. (S21)).

Supplementary Method 4. Axial position reconstruction of the rosette center

Noting the diffraction rings in the phase-contrast imaging (Supplementary Fig. **6**a), we identified a single cell using a similar ring pattern (Supplementary Fig. **6**b, inset), here the zeroth order Bessel function, i.e., $f_J(\rho) = J_0(k_1\rho/\rho_0)$, where $k_1 = 2.4048$ is the first root of $J_0(r)$, and $\rho_0 = 0.2$ μm is the estimated cell radius. A 2D autocorrelation with this Bessel-function filter was computed for the entire raw image to identify all individual cells (Supplementary

Fig. 6b). The filtered image is then subjected to Gaussian blur such that the rosette appears as a simply connected object (Supplementary Fig. 6c).

The contrast level of the filtered image is found to decrease monotonically with the offset of the rosette center from the focal plane, as expected from the decreased fraction of in-focus member cells. Such a contrast level is thus a unique function of the rosette's offset from the focal plane, which can be combined with the axial position of the microscope stage to determine the rosette's axial movement. The uncertainty of such a z estimation is approximately less than 3 μm, based on the fluctuation of the image contrast (Supplementary Fig. 6d). During the experiment, the center of the rosette is held above the focal plane, using a piezo stage (Physik Instrumente PInano XYZ High Dynamics Nanopositioning stage system), to maintain a one-to-one mapping between the axial (z) position and the contrast level (Supplementary Fig. 6d). However, this mapping is not universal and thus requires a calibration for each studied rosette (Supplementary Fig. 7), due to the potential variation in rosette sizes and cell concentrations.

Supplementary Method 5. Polarity analyses of the translation-rotation coupling angle β

To further understand how a nearby surface affects the translation-rotation coupling, we categorized β, the angle between in-plane translational ($\mathbf{u}_{x\text{-}y}$) and angular ($\boldsymbol{\omega}_{x\text{-}y}$) velocity (see Fig. 3a), into dimensionless gap sizes d/R. The corresponding probability distribution functions $\rho(\beta)$ for different d/R are shown in Supplementary Fig. 8a. To show whether there is any polarity in such a distribution, we fit each of these distributions (through standard MatLab nonlinear regression) by a single-period sinusoidal function (Supplementary Fig. 8b), i.e., $\rho(\beta) = 1/(2\pi) + \rho_0 \sin(\beta + \beta_0)$ with ρ_0 and β_0 as the fitting parameters. The polarity of these distributions is thus computed by the maxima $\rho_{\max}$ and minima $\rho_{\min}$ of their fitted values as $P = 2(\rho_{\max} - \rho_{\min})/(\rho_{\max} + \rho_{\min})$ or $P = 2\pi\rho_0$. The calculated values of P for various gap sizes are shown in the inset of Fig. 3b.

Supplementary Method 6. Hydrodynamic interactions in rosette rolling

The rotation-translation coupling of a microscale object in a Newtonian fluid can be obtained by solving the Stokes equation due to the negligible inertia of fluids. For a smooth sphere rotating and translating near a solid surface, its hydrodynamic force $\mathbf{F}$ and torque $\mathbf{L}$ are linearly proportional to the velocity $\mathbf{u}$ and angular velocity $\boldsymbol{\omega}$ through a resistance matrix [4], i.e.,

$$\begin{pmatrix} F_x \\ F_y \\ F_z \\ L_x \\ L_y \\ L_z \end{pmatrix} = \begin{pmatrix} -\mathcal{N}^{FU}_{xx} & 0 & 0 & 0 & \mathcal{V}^{F\Omega}_{xy} & 0 \\ 0 & -\mathcal{N}^{FU}_{yy} & 0 & -\mathcal{V}^{F\Omega}_{yx} & 0 & 0 \\ 0 & 0 & -\mathcal{N}^{FU}_{zz} & 0 & 0 & 0 \\ 0 & -\mathcal{V}^{LU}_{xy} & 0 & -\mathcal{N}^{L\Omega}_{xx} & 0 & 0 \\ -\mathcal{V}^{LU}_{yx} & 0 & 0 & 0 & -\mathcal{N}^{L\Omega}_{yy} & 0 \\ 0 & 0 & 0 & 0 & 0 & -\mathcal{N}^{L\Omega}_{zz} \end{pmatrix} \cdot \begin{pmatrix} u_x \\ u_y \\ u_z \\ \omega_x \\ \omega_y \\ \omega_z \end{pmatrix}, \tag{S23}$$

where the non-vanishing off-diagonal elements in the matrix correspond to the translation-rotation coupling. For a sphere (of radius R) subjected to external torque along the y axis, a force-free condition can be achieved by a

superposition of a non-rotating sphere that translates in the x direction and a non-translating sphere that rotates about the y axis. This superposition leads to a ratio of the translational speed u_x and linear rotation speed $\omega_y R$, or the slipping ratio $Q = u_x/(\omega_y R)$, as

$$Q = \mathcal{V}^{F\Omega}_{xy} / \left(\mathcal{N}^{FU}_{xx} R \right). \tag{S24}$$

For small sphere-wall gaps, i.e., $d \ll R$, an asymptotic behavior of the slipping ratio Q can be obtained through the lubrication-theory approximation [4] such that

$$\mathcal{N}^{FU}_{xx} = 6\pi\mu R \left[-\frac{8}{15} \ln\left(\frac{d}{R}\right) + 0.96 \right], \tag{S25}$$

and

$$\mathcal{V}^{F\Omega}_{xy} = 6\pi\mu R^2 \left[-\frac{2}{15} \ln\left(\frac{d}{R}\right) - 0.25 \right], \tag{S26}$$

where μ is the kinetic viscosity of the fluid. The asymptotic dependence of Q on R/d due to hydrodynamic forces is thus

$$Q = \frac{\frac{2}{15} \ln\left(\frac{d}{R}\right) + 0.25}{\frac{8}{15} \ln\left(\frac{d}{R}\right) - 0.96}. \tag{S27}$$

It can be shown that index Q approaches $1/4$ for vanishing d and decreases monotonically for relatively small d ($0 < d \lesssim 0.153R$). For $d \gtrsim 0.153R$, the asymptotic Q becomes negative and the lubrication approximation no longer holds.

To extend the evaluation of the hydrodynamic effects on rolling for finite gap sizes d, we computed the resistance matrix elements and thus the Q values through a half-space boundary integral method (BIM) [5]. Here, the smooth sphere is modeled as a frequency-24 icosahedron (with 11520 triangularized facets). The numerical algorithm has been validated by comparing the computed resistance matrix elements with the analytic results through multipole expansions [6] (Supplementary Table **1**).

The asymptotic Q and that computed by the BIM are both shown in Supplementary Fig. **9**. The BIM results (red) approach the asymptotic values (blue) as d decreases, agreeing with the predictions from the lubrication theory. In both computations, the hydrodynamic effects alone can only lead to a maximum slipping ratio of 0.25 when d/R is essentially zero, inadequate to compensate for the high slipping ratio observed for rosettes at finite d/R. Other mechanisms, e.g. physical contact, may play a significant role in rosette rolling.

Supplementary Method 7. Statistical tests of the slipping ratio Q

To examine the statistical significance of the slipping ratio (Q) data, we adopted two types of statistical test: regression and pairwise comparison.

Before performing the regressions, we averaged the data (originally collected at 20 Hz) over every one second to filter out high-frequency noises. However, it can be shown that the regression trend is indeed insensitive to the exact

sampling rate chosen by us. For the regression test, we applied both a linear regression model to data with relatively small gap sizes ($d/R < 2$) and a nonlinear regression to all data. Notably, the model that we used for nonlinear regression was an exponential function with arbitrary offset, i.e., $Q(d/R) = c_1 + c_2 e^{-c_3 d/R}$ with c_1, c_2, and c_3 as the fitting parameters. Both the linear and nonlinear regressions show similar intercepts, $Q = 0.56 \pm 0.10$ (mean ± 95% confidence interval) vs. $Q = 0.56 \pm 0.13$ (mean ± 95% confidence interval), which is significantly higher than the maximum value predicted by the hydrodynamic model (with a smooth sphere), i.e., $Q = 0.25$ for $d/R = 0$ (Supplementary Fig. **10**a). These regression tests were performed by the standard regression package in MatLab.

For pairwise comparisons, we grouped the data based on d/R intervals of $0.5(d/R)$ and used one-way analysis of variance (ANOVA) to test the significance of Q variation due to gap size. We then performed pairwise comparison with the Tukey post-hoc test using function 'tukey_hsd' in package 'rstatix' written in R [7]. The comparison result is shown in Supplementary Fig. **10**b. The slipping ratio Q for the smallest d/R group ($d/R < 0.5$) is significantly different from that for higher d/R groups ($d/R > 0.5$).

Supplementary Method 8. Rosette and solitary-cell kinematics

Both the *C. crescentus* solitary cells and rosettes were observed under the microscope for extended durations by automatically and manually adjusting the 3D microscope stage to follow the targets, respectively [1]. The typical trajectories of a solitary cell and a rosette are shown in Supplementary Fig. **2**a, which exhibit distinct characteristics. The trajectory of a solitary cell is composed of consecutive linear segments forming alternating acute and right angles, corresponding to the run-reverse-flick movements of monotrichous bacteria [8, 9]. In contrast to the solitary case, the trajectory of a rosette is composed of erratically curved segments. These segments either follow a clockwise (CW) or a counter-clockwise (CCW) circulation (as viewed from the top). In addition to the geometries in trajectories, the speeds of a solitary cell and a rosette are also distinct. As shown in histograms (Supplementary Fig. **2**b), the average speed of a solitary cell is 45±19 μm/s (mean±S.D. from 119 cells), which is more than an order of magnitude faster than that of a rosette, 3.6±2.3 μm/s (mean±S.D. from 71 rosettes). While the probability distribution of the speed for a solitary cell is Gaussian-like, that distribution has a noticeable positive skewness for a rosette. We conjecture that this skewness is associated with the period of waiting for a newborn cell to propel the rosette.

For rosettes moving near the solid surfaces, we also characterized their kinematics by the turning angles between adjacent motor switches and compared the outcome with the solitary case. These turning angles are determined by the abrupt changes of the moving directions in a quasi-2D trajectory. Consecutive turning angles for various trajectories are shown in Supplementary Fig. **11**. In the solitary case (Supplementary Fig. **11**a), the turning angle alternates between an obtuse angle close to π (corresponding to a reverse) and a moderate angle close to $\pi/2$ (corresponding to a flick). In the rosette case (Supplementary Fig. **11**b), the turning angle is mostly obtuse, independent of the motor directions. The running direction of the flagellar motor for the rosette is obtained by identifying the rotation direction of the rosette and the direction of circulation in its trajectory.

The statistics of the motor activities in solitary cells and rosettes are shown side-by-side for comparison (Supple-

mentary Fig. **12**). In both cases, the motor spent almost the same amount of time in CW and CCW directions (slightly longer in the CW direction). The mean durations and their standard deviations are overall greater in the rosette case, reflecting the relative heavier tails in the distribution functions (Fig. 4c). The crossover times obtained from their mean-squared displacement curves (Fig. 1c) are also shown (in open circles) for reference, which are 1.29 ± 0.03 s and 1.18 ± 0.26 s for the solitary cell and the rosette, respectively.

Supplementary Method 9. Segmented active regimes in long-term rosette dispersal

While this study focuses on the active propulsion by flagellar motors, it is worth noting that the entire dispersal of rosettes cannot be attributed solely to this active regime, presumably sophisticated because of the dynamic nature of cell divisions and its pertinence to a rosette's powering sources. As exemplified in Supplementary Fig. **13**a, the long-term ($\gtrsim$ 10 min) dispersal of a rosette was found to consist of a series of a "motile" and an "almost immotile" segments, signified by their distinct speeds (shown as probability distribution functions in Supplementary Fig. **13**b). The finite duration of each consecutive motile regime (~ 1 min) is consistent with the origin of the motile organelles: each stalked cell member contributes to rosette motility through a flagellated daughter cell during its division (totally lasting for ~ 100 min [10, 11]). We argued that these consecutive motile regimes were associated with individual sets of flagella and motors since there were no obvious chemical or mechanical cues to suppress all motors for such a considerably long duration ($\gtrsim 1$ min), other than detachment of those previously flagellated daughter cells. We therefore regard the duration of these motile regimes an indicator of how long a daughter cell is flagellated before its detachment, here $\Delta t_f \sim 1$ min (Supplementary Fig. **13**).

Supplementary Method 10. Statistical model for the number of functioning motors in a rosette

As another limiting factor for the number of available flagellar motors, the size of the rosettes formed under our protocol was narrowly distributed ($R = 4.0 \pm 1.2$ μm; mean $\pm$ S.D.; Supplementary Fig. **1**), corresponding to an estimate of $N_s \approx 15$–25 stalked cells. In the following, we argue that this finite N_s potentially underlies the single-motor powering of rosettes in this study. Based on the life cycle of *C. crescentus*, each predivisional cell within a rosette hosts a functioning flagellum for a duration Δt_f shorter than the total division time Δt_d, lagged by the necessary growth time for the motile organelle. We can thus define the probability of finding a flagellated predivisional cell by $P_f = \Delta t_f / \Delta t_d$. Also, over a long time scale far beyond the aforementioned ballistic regime (~ 1 s), the active dispersal of a rosette was frequently interrupted by long pauses (lasting up to 10 min), over which the rosette exhibited a considerably lower motility (see Supplementary Fig. **13** and Movie S1). It is plausible that such a transition to low motility is due to the detachment of a daughter cell. In this sense, the pausing events can be used to estimate a timescale for the flagellated duration Δt_f, with the assumption that each terminated motor can no longer be employed by the rosette for dispersal (due to, for instance, a cell detachment). A continuous powering duration between two adjacent pausing events thus provides an order-of-magnitude estimate for the flagellated duration Δt_f, i.e., $\Delta t_f \sim 1$ min (Supplementary Fig. **13**). Combined with a division timescale, $\Delta t_d \sim 10^2$ min [10, 11], we

estimated that the flagellated probability for each stalked member $P_f = \Delta t_f / \Delta t_d \sim 10^{-2}$. Given the typical number of member cells ($N_s \lesssim 25$) of a rosette in this study and the assumption that the divisions of all members are independent, we achieved an expected number of flagellated cells (n) for a rosette (with a total N_s cells) to be $E(n) = N_s p_f \lesssim 1$. Further, the probability for finding n flagellated cells simultaneously shares the same solution with a classic coin flipping problem, as given by a binomial distribution formula $P(n, N_s) = \binom{N_s}{n} P_f^n (1 - P_f)^{N_s - n}$. Considering a relatively large rosette size (likely to give rise to more flagellated cells) in this study, i.e., $N_s = 25$, and $P_f = 10^{-2}$ (from the above order-of-magnitude estimate), we have the probabilities for finding null and one flagellum to be $P(0, 25) = 0.778$ and $P(1, 30) = 0.196$, respectively. The probability for more than one flagellated cell is thus $P(>1, 25) = 1 - P(0, 25) - P(1, 25) = 0.026$, which is almost an order of magnitude lower than the probability for a single flagellated cell $P(1, 25)$. All these results are consistent with the apparent single-motor driving observed in our experiments, suggesting a potential mechanism of regulating the powering source of rosettes by the amount and the division rate of member cells. It is also worth noting that the above analysis does not exclude the possibility that the lack of motility in rosette culture long-sitting on glass slides was associated with other unknown experimental variables, such as temperature (e.g., a room temperature $\approx 24°$C, considerably lower than rosettes' culture temperature $\approx 30°$C).

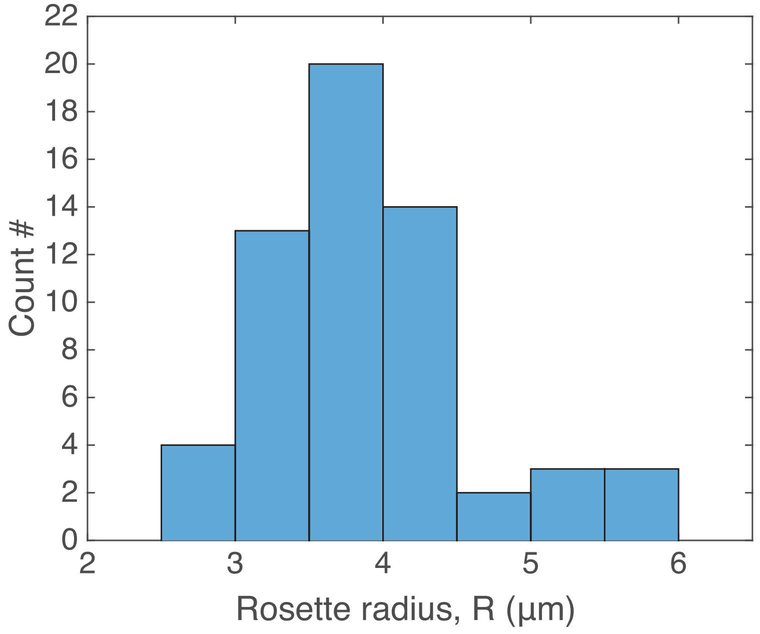

Supplementary Figure 1: **Histogram of rosette size.** The radii R of rosettes in this study range from 2 to 6 μm, with its distribution peaks around 4 μm.

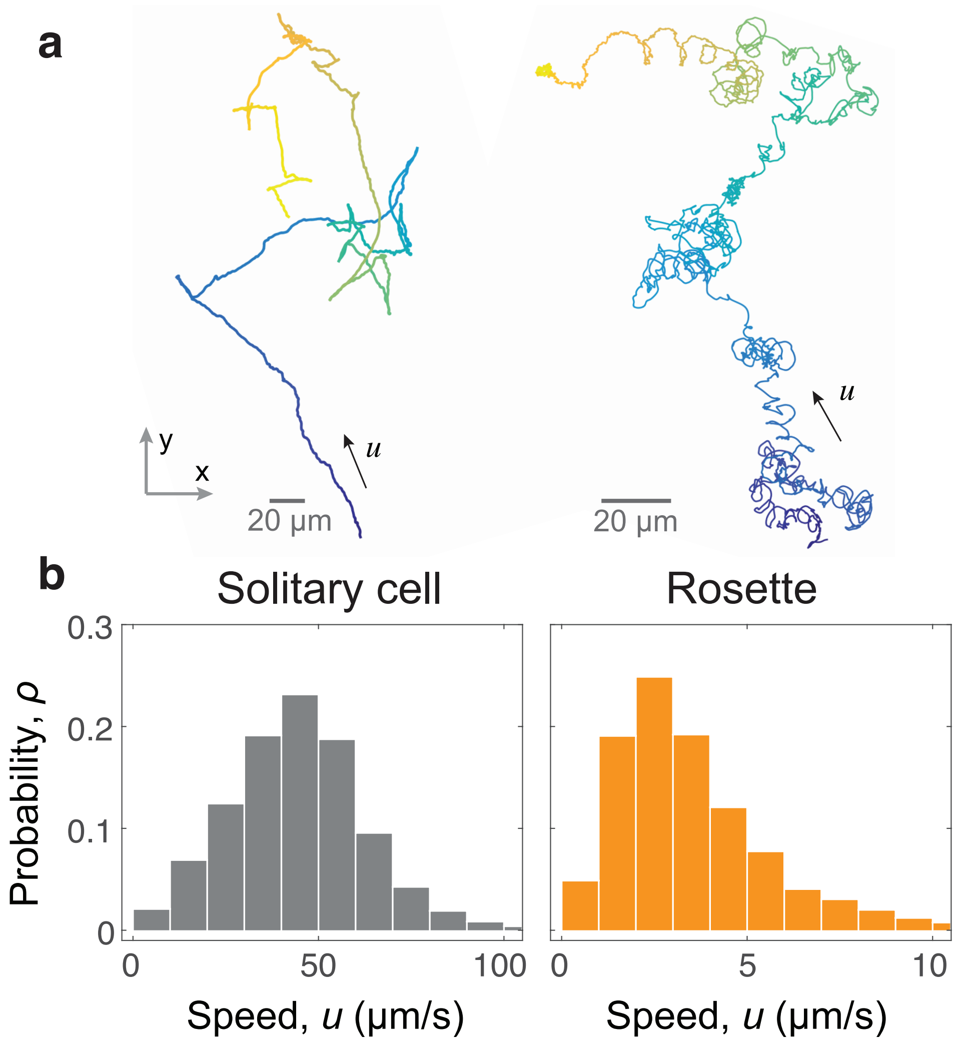

Supplementary Figure 2: **Transport of rosettes and solitary cells. a** Typical trajectories for a solitary cell (left) and a rosette (right), color-coded in time. **b** Histograms of the transporting speed for a solitary cell (left) and a rosette (right). All speed statistics are sampled for $\gtrsim 1$ s for individual swarmer cells and rosettes (at 160 Hz for swarmer cells and at 20 Hz for the slower-swimming rosettes). The numbers of data points are $N = 70629$ from $N = 119$ cells and $N = 5295$ from $N = 71$ rosettes.

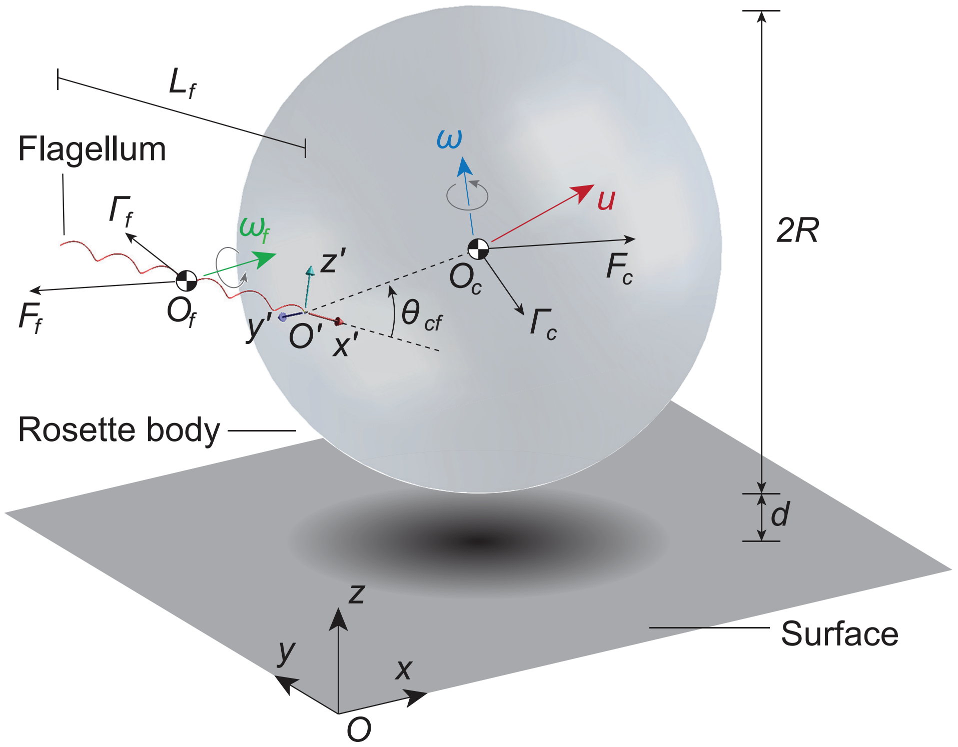

Supplementary Figure 3: **Schematic of the hydrodynamic model of an actively propelling rosette**. The rosette is considered near a wall that lies in the x-y plane of a lab frame of reference (x, y, z). The rosette geometry is estimated as a smooth sphere (to represent all cell bodies aggregated within the rosette) of radius R and a finite helical filament [2] of the same dimension of the flagellum [12] possessed by a swarmer cell. The spherical cell aggregate and the helical flagellum are centered at O_c and O_f, respectively and intersect at O'. Their combined dynamics are examined by a resistive-force-type approach [1], with the forces $\mathbf{F}$ and torques $\boldsymbol{\Gamma}$ on both objects (sphere and helix) solved independently and satisfying the force- and moment-balance conditions. In this model, a body-fixed frame of reference (x', y', z') is conveniently defined by aligning the x' axis along the helical axis of the flagellum and confining the separation vector between the aggregate and the flagellum ($\overrightarrow{O_c O_f}$) in the x'-z' plane. The deviation of the flagellar axis from the radial direction is accounted for by introducing an alignment angle θ_{cf}, the angle between the vector $\overrightarrow{O'O_c}$ and the x' axis. The angular velocity of the helix $\boldsymbol{\omega}_f$ relative to the sphere $\boldsymbol{\omega}$ is prescribed in the body-fixed frame of reference, i.e., $\boldsymbol{\omega}_0 = \boldsymbol{\omega}_f - \boldsymbol{\omega} = \omega_0 \hat{x}'$, with ω_0 and $\hat{x}'$ denoting a fixed motor speed and a unit vector along the x' direction, respectively. The hydrodynamic interaction between sphere and wall (with a gap d) can be represented by a resistive matrix computed by the above BIM. The interaction between flagellum and wall, however, is neglected here due to their typical large separations.

[htb]

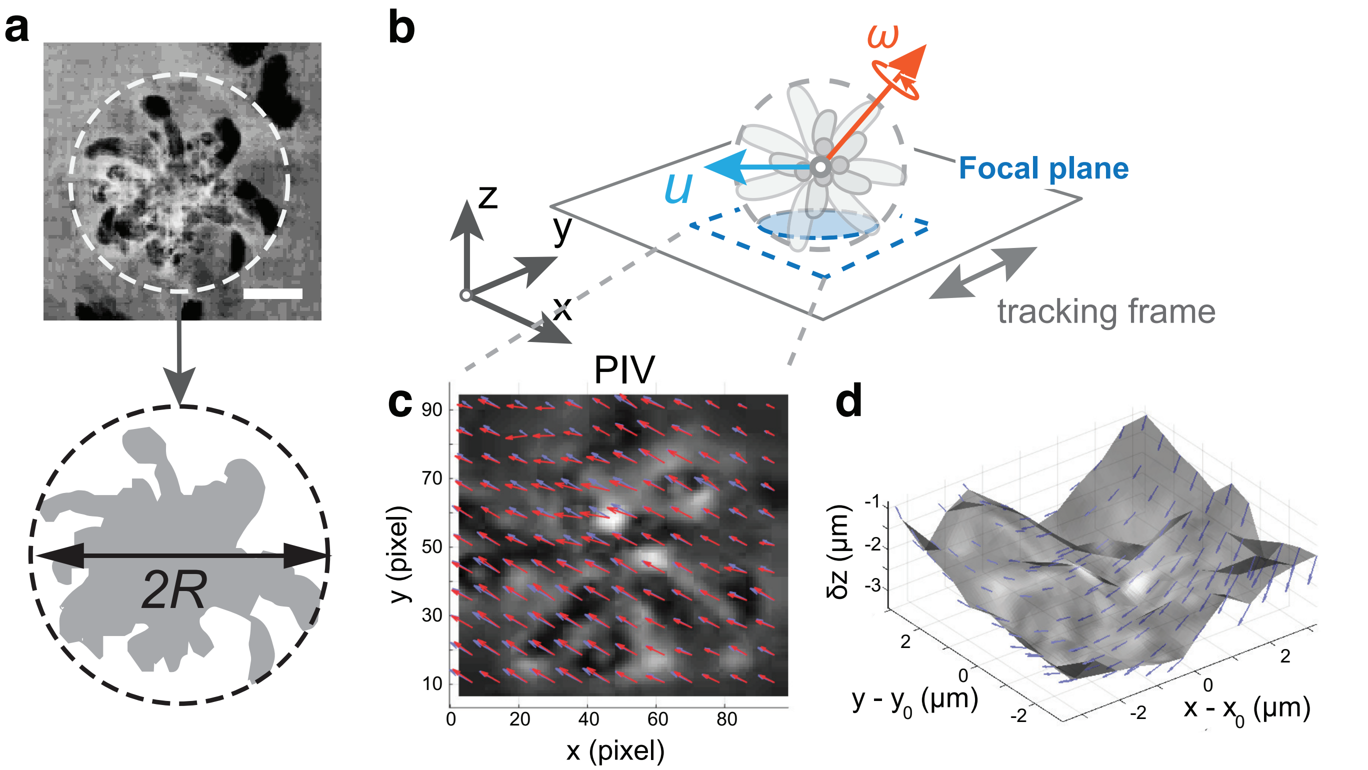

Supplementary Figure 4: **Rosette rotation reconstruction. a** The size (or radius *R*) of the rosette are determined by finding a minimal enclosing circle of its projected area (bottom) obtained from *z* stacks of its phase-contrast image (top). Scale bar, 2 μm. **b** A schematic drawing of rotation reconstruction of a rosette from its velocity field near the focal plane. **c** The lateral velocity field (u_{x-y}) of the rosette near the focal plane is obtained from the PIV analysis, shown in red arrows. The best-matching velocity field due to a 3D rigid-body rotation is shown in blue arrows. **d** The same best-matching velocity field is shown in a side view. The fluctuating axial position (δz) of the imaged plane is reconstructed from Eq. (S20).

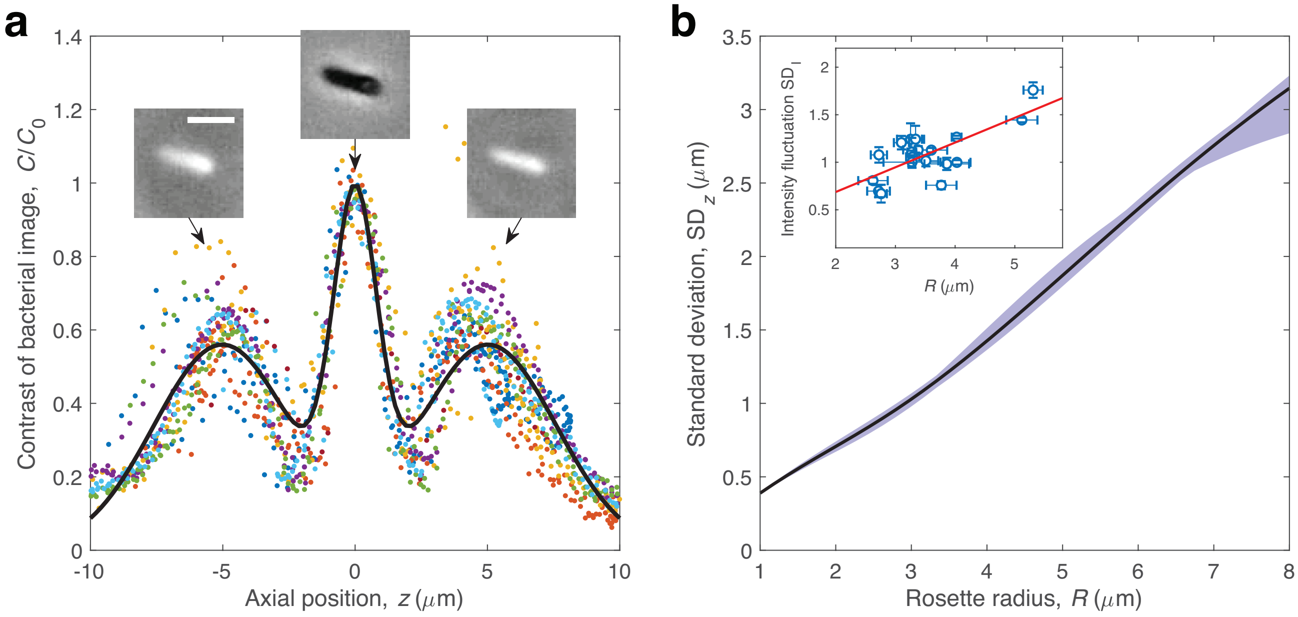

Supplementary Figure 5: **Characterization of the *z*-variation in a rosette image. a** The contrast level of a single-cell image with varying offset from the focal plane (z). Dots of different colors correspond to different individual bacteria (total 13) used in this measurement, which can be well fit by a sum of three gaussian distribution (solid curve). The insets show the images of a single cell corresponding to the peak contrast levels. Scale bar, 2 μm. **b** The standard deviation of the axial position SD_z for varying rosette radius R, as computed by Eq. (S22) and based on the previously obtained contrast profile (solid curve shown in **a**) with the shade denoting the uncertainty. The inset shows the experimental measurement of the standard deviation of the image intensity SD_I for various rosettes (with R varying from 2.5 to 5.2 μm).

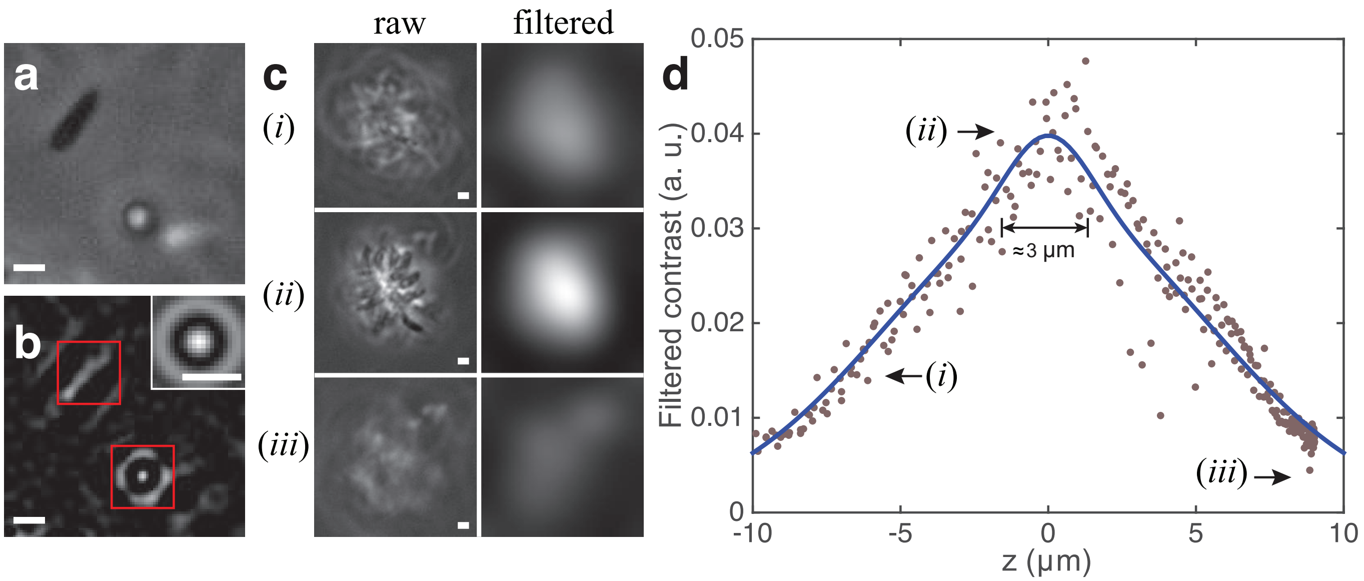

Supplementary Figure 6: **Rosette axial position calibration. a** Example images of individual cells under the phase-contrast imaging. **b** The image of cells (shown in **a**) subjected to a Bessel-function filter (inset). **c** Examples of the filtered rosette images (through a Bessel-function filter and a Gaussian blur) and corresponding raw frames captured at different focal planes (i-iii, as labeled in **d**). **d** The contrast level of a filtered rosette image as a function of the axial position (z). The dots show the experimental data while the solid curve shows a polynomial fit. The typical uncertainty of the axial position is less than 3 μm, which peaks at $z = 0$. Scale bars, 1 μm.

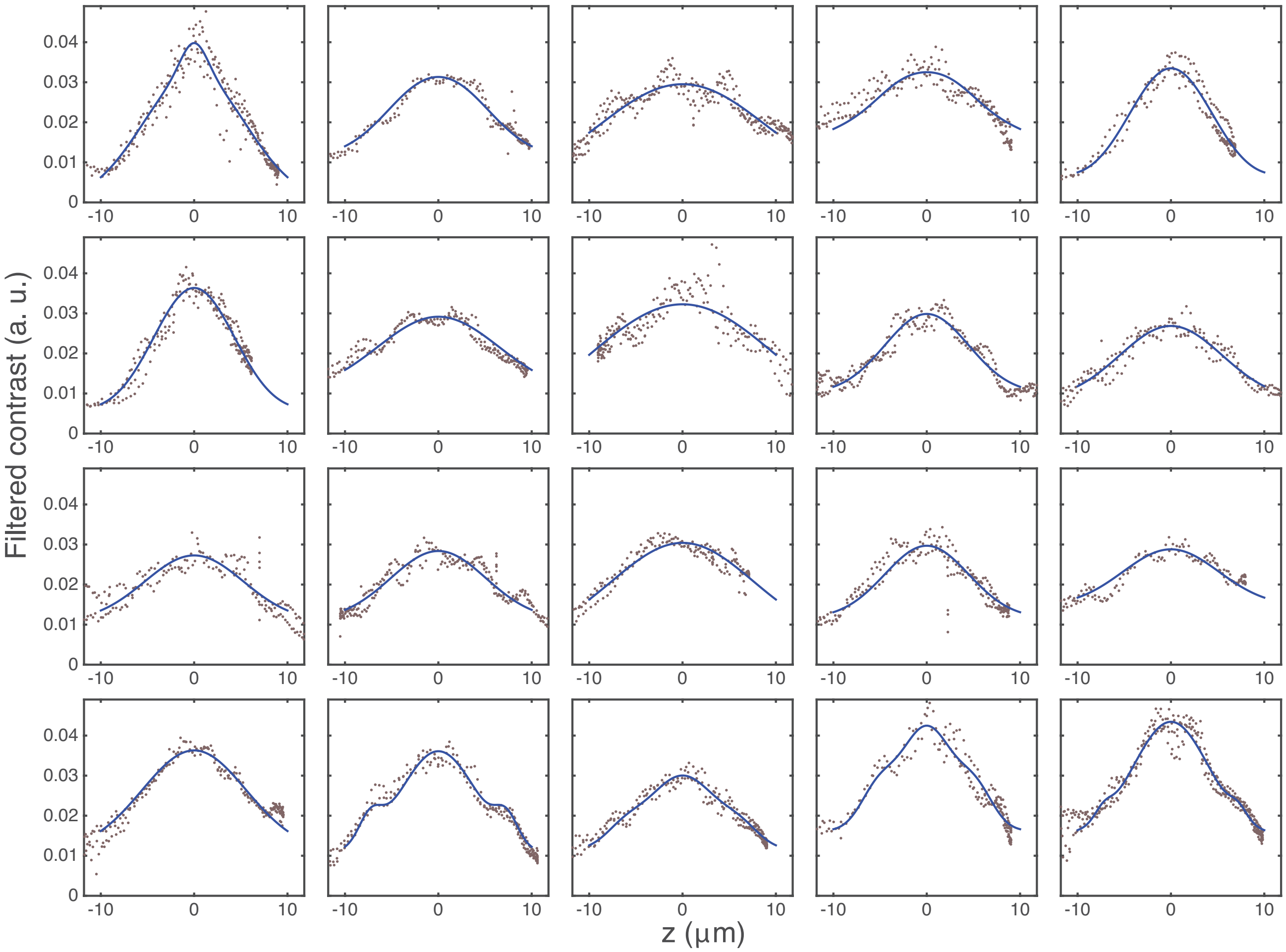

Supplementary Figure 7: **Variation of contrast level vs. axial position curves among rosettes.** Each panel corresponds to the measurement of the filtered contrast level of an individual rosette, as a function of its axial offset from the focal plane, which is used for axial-position reconstructions.

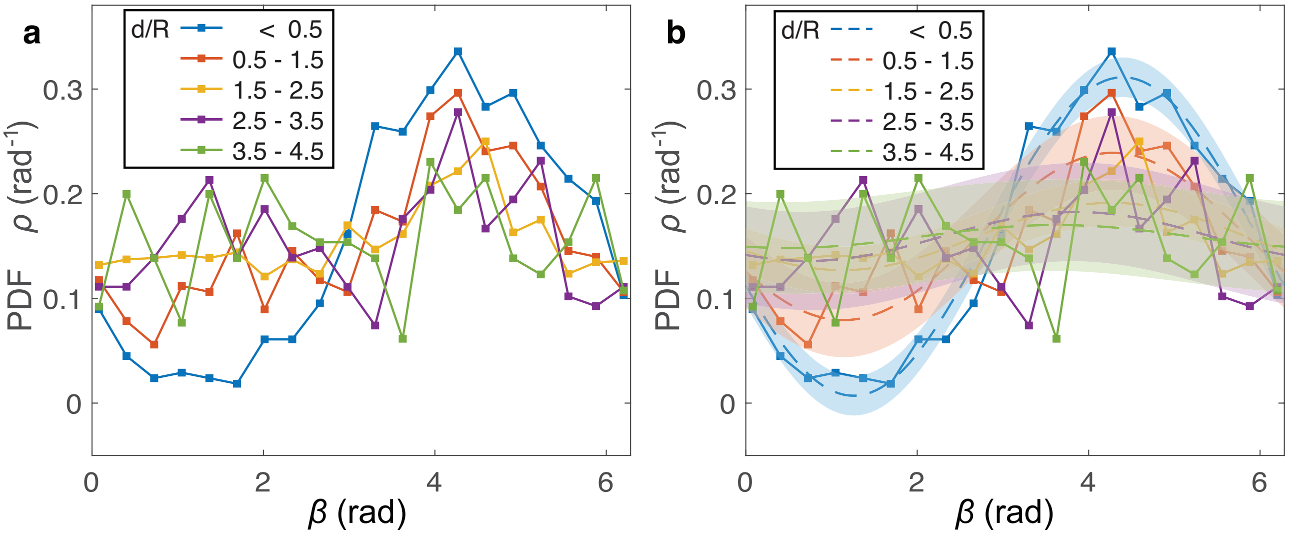

Supplementary Figure 8: **Dependence of the rotation-translation coupling angle on gap size. a** Probability distribution functions ρ of the angle between in-plane translation and rotation, β, for varying gap size d (normalized by rosettes' radii R). **b** Each probability distribution function in **a** was fit by a sinusoidal function (dashed line) for computing the polarity $P = \frac{2(\rho_{\max}-\rho_{\min})}{\rho_{\max}+\rho_{\min}}$, where $\rho_{\min}$ and $\rho_{\max}$ are minimum and maximum of function ρ, respectively. Shaded areas show the 95% confidence bounds of the fitting.

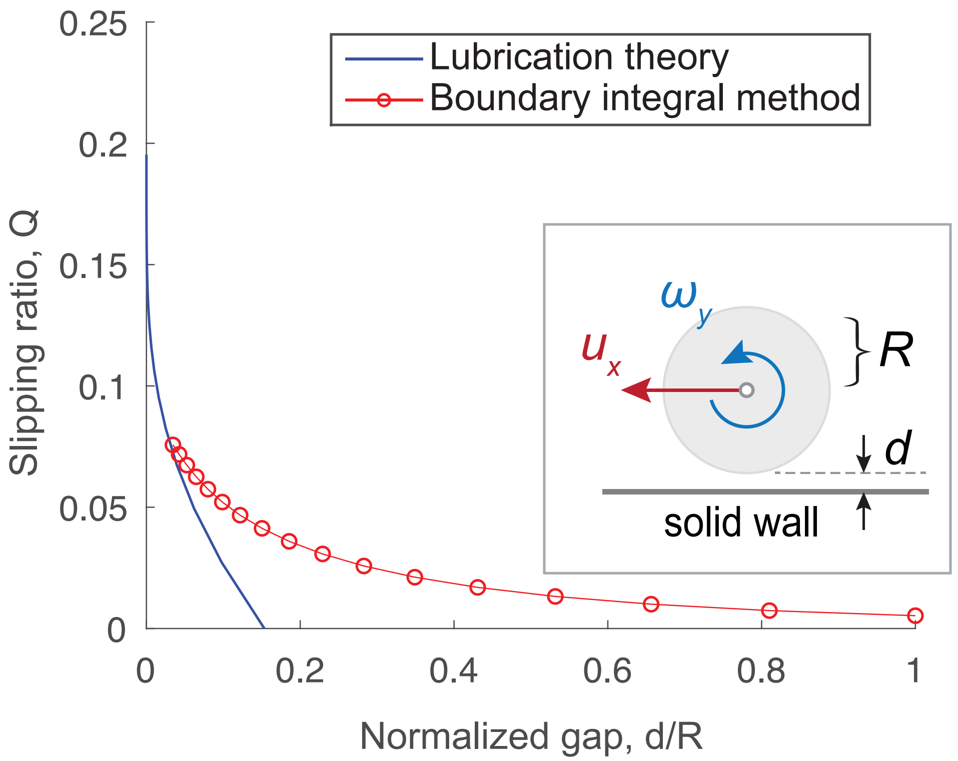

Supplementary Figure 9: **Computed slipping ratio based on hydrodynamic interactions.** Blue line is the asymptotic result based on the lubrication theory (Eq. (S27)). Red line with open circles corresponds to the simulated results using a half-space boundary integral method (Ref. [5, 13]).

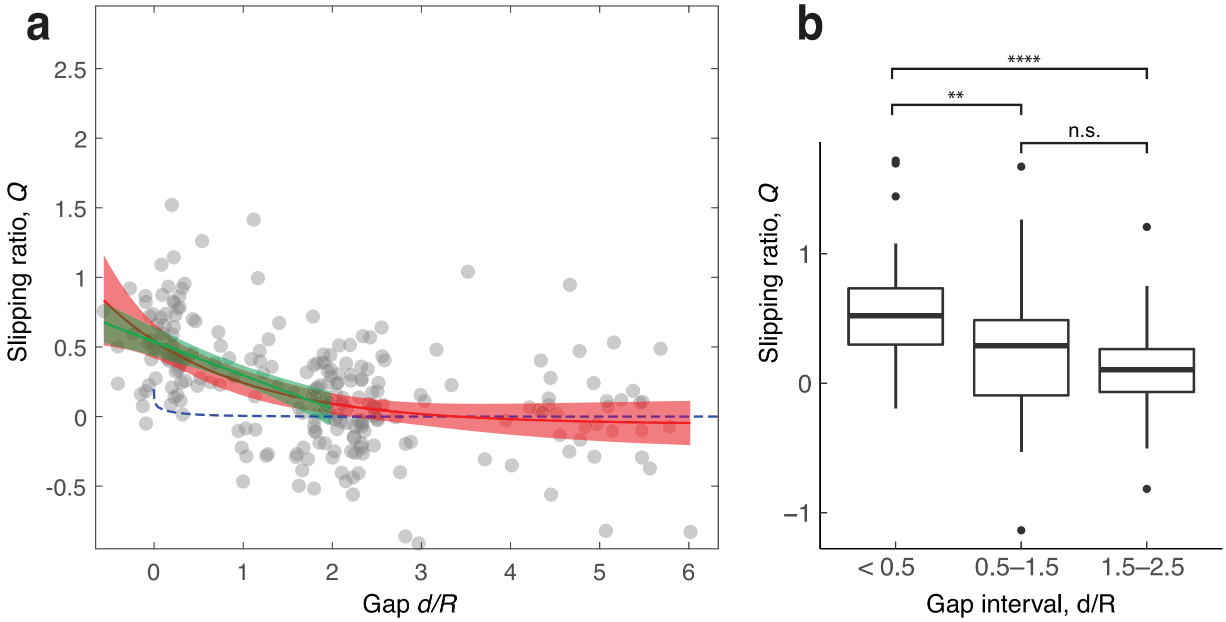

Supplementary Figure 10: **Statistical tests for the slipping ratio Q as a function of the dimensionless gap size d/R. a** Both a linear regression (for $d/R < 2$; green) and a nonlinear regression (for all d/R; red) were applied to the experimental data (filled circles; averaged over every one second). The shaded areas correspond to the 95% confidence bounds of the regressions (red: nonlinear, green: linear). The result for the hydrodynamic model (dashed line) reaches the maximum ($Q = 0.25$) at $d/R = 0$. **b** Variation of Q with respect to gap size (d/R) groups. Box plots are based on mean values calculated over 1 s time intervals. Values are significantly different among gap size groups (one-way ANOVA, F2,141 = 19.07, $p < 0.0001$). Asterisk symbol denotes the values significantly different between gap intervals in pairwise comparisons following one-way ANOVA (**, $p < 0.01$; ****, $p < 0.0001$; n.s., $p > 0.05$).

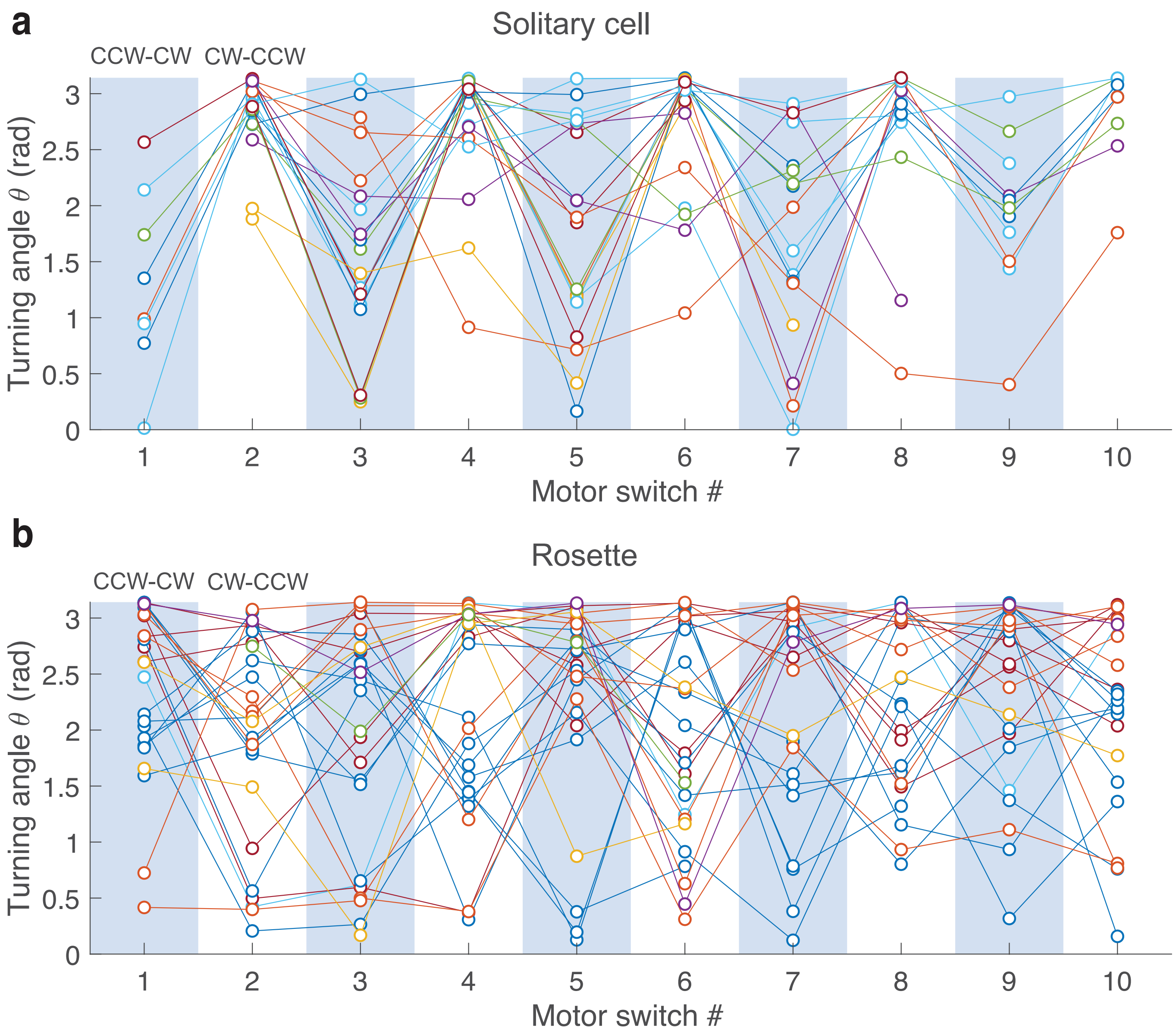

Supplementary Figure 11: **Turning angles due to motor switches for solitary cells and rosettes. a** Turning angles for *C. crescentus* solitary cells (16 consecutive tracks). **b** Turning angles for *C. crescentus* rosettes (20 consecutive tracks).

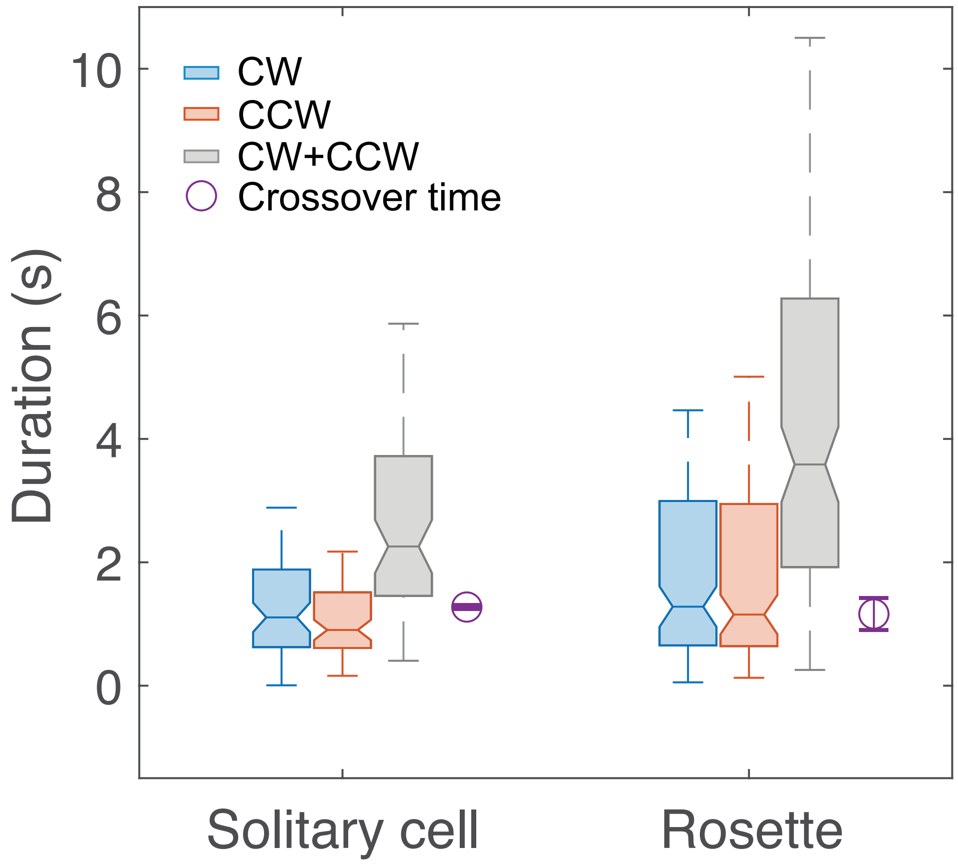

Supplementary Figure 12: **Motor kinematics in the solitary cell and the rosette case.** The durations of the flagellar motor state in each rotation direction (CW and CCW), and its restoring time (CW + CCW) are shown in box plots for both the solitary cells and the rosettes. The open circles correspond to the crossover time (Fig. 1) with the error bars showing their standard errors.

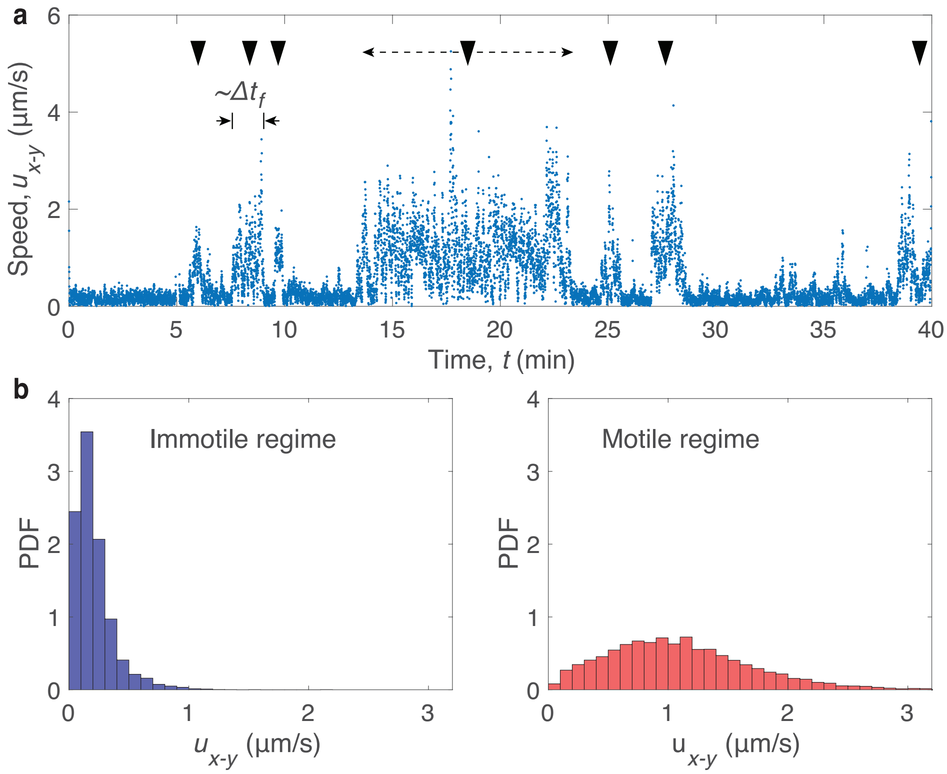

Supplementary Figure 13: **Segmented motile regimes in rosette's long-term dispersal. a** A long-term recording (for over 40 min) of the speed of an individual rosette in the image plane ($u_{x\text{-}y}$) exhibited discrete motile regimes (with $u_{x\text{-}y} \sim 1$ μm/s), interrupted by those immotile regimes with much reduced speed (with $u_{x\text{-}y} \sim 0.1$ μm/s). The duration of these continuous motile regimes serves as an indicator for the duration of a set of functioning motors Δt_f achieved through flagellated daughter cells. An extremely long motile regime (e.g., the high-speed zone near the center, highlighted by dashed arrows) potentially contains several discrete motile regimes (driven by multiple sets of motors) without noticeable gaps. **b** The probability distribution functions (PDF) of $u_{x\text{-}y}$ in these two regimes (in **a**) signify their characteristic speeds, which are significantly different: 1.11 ± 0.62 μm/s (motile) v.s. 0.21 ± 0.16 μm/s (immotile).

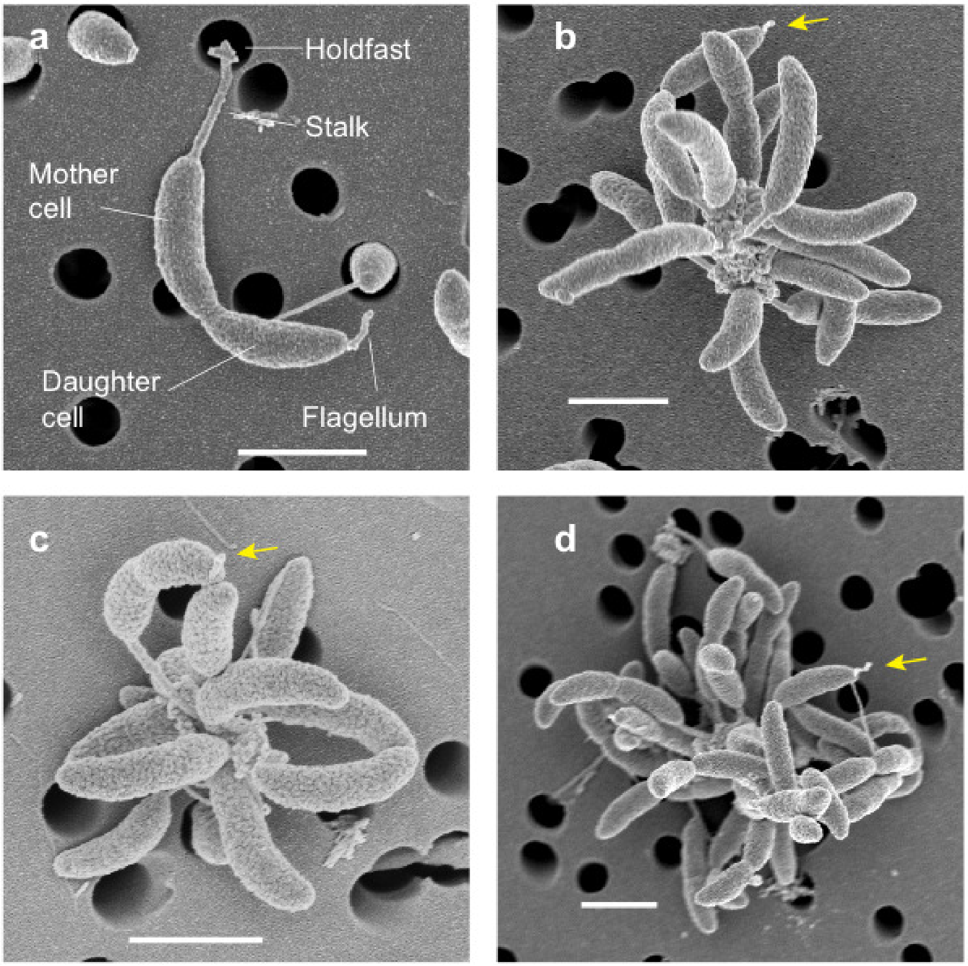

Supplementary Figure 14: **Scanning electron microscopy (SEM) images. a** An image of a typical predivisional *C. crescentus* cell, showing the asymmetric cell devision between a mother (or stalked) and a daughter (or swarmer) cells. The stalk grown on the stalked end may potentially attach to a adhesive mass of holdfast shared with other stalked cells to form a rosette. The flagellum grown on the daughter cell may potentially be employed by the rosette for self propulsion. **b**–**d** Images of *C. crescentus* rosettes under our culture protocols, showing the radial arrangement of stalked cells attached to a core. Arrows indicate partially damaged flagella due to SEM preparation. Scale bars, 1 μm.

Supplementary Table 1: Examples of the elements of the resistance matrix computed by the half-space boundary integral methods, as compared to the multipole-expansion results accurate to four significant figures (in brackets) according to Ref. [6].

d/R	$\mathcal{N}_{xx}^{FU}/(6\pi\mu R)$	$\mathcal{N}_{zz}^{FU}/(6\pi\mu R)$	$\mathcal{V}_{xy}^{F\Omega}/(8\pi\mu R^2)$
2.7622	1.1744 [1.1738]	1.4137 [1.4127]	0.00042163 [0.0042161]
1.3524	1.3086 [1.3079]	1.8391 [1.8371]	0.0026409 [0.0026424]
0.5431	1.5687 [1.5675]	3.0422 [3.0360]	0.014613 [0.014649]
0.1276	2.1567 [2.1515]	9.4778 [9.2516]	0.072416 [0.073718]

Supplementary References

[1] Liu, B. *et al.* Helical motion of the cell body enhances *Caulobacter crescentus* motility. *Proceedings of the National Academy of Sciences of the United States of America* **111**, 11252–11256 (2014).

[2] Liu, B. & Gonzalez, J. Bundled slender-body theory for elongated geometries in swimming bacteria. *Physical Review Fluids* **5**, 053102 (2020).

[3] Thielicke, W. & Stamhuis, E. J. PIVlab – towards user-friendly, affordable and accurate digital particle image velocimetry in MATLAB. *Journal of Open Research Software* **2**, e30 (2014).

[4] Lauga, E., DiLuzio, W. R., Whitesides, G. M. & Stone, H. A. Swimming in circles: Motion of bacteria near solid boundaries. *Biophysical Journal* **90**, 400–412 (2006).

[5] Gimbutas, Z., Greengard, L. & Veerapaneni, S. Simple and efficient representations for the fundamental solutions of Stokes flow in a half-space. *Journal of Fluid Mechanics* **776** (2015).

[6] Ozarkar, S. S. & Sangani, A. S. A method for determining Stokes flow around particles near a wall or in a thin film bounded by a wall and a gas-liquid interface. *Physics of Fluids* **20**, 063301 (2008).

[7] Kassambara, A. *Rstatix: Pipe-Friendly Framework for Basic Statistical Tests* (2020).

[8] Xie, L., Altindal, T., Chattopadhyay, S. & Wu, X.-L. Bacterial flagellum as a propeller and as a rudder for efficient chemotaxis. *Proceedings of the National Academy of Sciences of the United States of America* **108**, 2246–2251 (2011).

[9] Son, K., Guasto, J. S. & Stocker, R. Bacteria can exploit a flagellar buckling instability to change direction. *Nature Physics* **9**, 494–498 (2013).

[10] Degnen, S. T. & Newton, A. Chromosome replication during development in *Caulobacter crescentus*. *Journal of Molecular Biology* **64**, 671–680 (1972).

[11] Iyer-Biswas, S. *et al.* Scaling laws governing stochastic growth and division of single bacterial cells. *Proceedings of the National Academy of Sciences* **111**, 15912–15917 (2014).

[12] Koyasu, S. & Shirakihara, Y. Caulobacter crescentus flagellar filament has a right-handed helical form. *Journal of Molecular Biology* **173**, 125–130 (1984).

[13] Liu, B., Breuer, K. S. & Powers, T. R. Helical swimming in Stokes flow using a novel boundary-element method. *Physics of Fluids* **25**, 061902 (2013).
