# Supplementary material for: Self-propelling and rolling of a sessile-motile aggregate of the bacterium Caulobacter crescentus
Source: Commun Biol. 2020 Oct 16;3:587. doi: 10.1038/s42003-020-01300-w (PMC7568532; doi:10.1038/s42003-020-01300-w)
Supplement: Supplementary file 2 — Description of Additional Supplementary Files [file 42003_2020_1300_MOESM2_ESM.pdf]

Description of Additional Supplementary Files

File Name: Supplementary Movie 1.
Description: Dispersion of *C. crescentus* rosettes over 10 minutes, which are highlighted by circles with their trajectories shown in colored lines. The view size is 152 μm × 152 μm.

File Name: Supplementary Movie 2.
Description: The quasi-2D velocity field of a moving rosette near the focal plane. The red arrows show the velocity field obtained from the particle image velocimetry (PIV), while the blue arrows correspond to the reconstructed velocity field of a rigid-rotating body. The view size is 47μm × 36μm.

File Name: Supplementary Movie 3.
Description: The full 3D (translational and rotational) kinematics of a rosette (of radius R = 4.0 µm) near the solid surface (with gaps d = 1.2±0.7 μm) are reconstructed from its phase-contrast images. The view size is 47μm × 36μm. A zoomed-in view of the rosette is overlaid with the 3D model for comparisons.

File Name: Supplementary Movie 4.
Description: The full 3D (translational and rotational) kinematics of a rosette (of radius R = 3.8 µm) relatively far from the surface (with gaps d = 19.9 ± 1.5 μm) are reconstructed from its phase-contrast images. The view size is 47μm × 36μm. A zoomed-in view of the rosette is overlaid with the 3D model for comparisons.

File Name: Supplementary Movie 5.
Description: Hydrodynamic simulation of a spherical rosette (of radius R = 3 µm) moving near a solid surface, with an initial gap size d = 1 μm and a swimming direction parallel to the surface. The flagellar axis is set radial, denoted by a 3D arrow.

File Name: Supplementary Movie 6.
Description: Hydrodynamic simulation of a spherical rosette (of radius R = 3 µm) moving near a solid surface, with an initial gap size d = 1 μm and a swimming direction parallel to the surface. The flagellar axis is set orthoradial, denoted by a 3D arrow.

File Name: Supplementary Movie 7.
Description: The reorientation (or the motor-switch) events of a rosette are identified with its trajectory color-coded by the running directions of the flagellar motor: blue for the clock-wise (CW) motor and red for the counter-clock-wise (CCW) one. The view size is 47μm × 36μm. A zoomed-in view of the rosette is shown in the inset.
